# Predictive and prognostic role of peripheral blood eosinophil count in triple-negative and hormone receptor-negative/HER2-positive breast cancer patients undergoing neoadjuvant treatment

**DOI:** 10.18632/oncotarget.26120

**Published:** 2018-09-14

**Authors:** Concetta Elisa Onesti, Claire Josse, Aurélie Poncin, Pierre Frères, Christophe Poulet, Vincent Bours, Guy Jerusalem

**Affiliations:** ^1^ Laboratory of Human Genetics, GIGA Research Institute, University of Liège, Liège, Belgium; ^2^ Department of Medical Oncology, University Hospital (CHU Liège), Liège, Belgium; ^3^ Laboratory of Medical Oncology, GIGA Research Institute, University of Liège, Liège, Belgium; ^4^ Department of Human Genetics, University Hospital (CHU Liège), Liège, Belgium

**Keywords:** eosinophil, neoadjuvant therapy, pathological complete response, triple-negative breast cancer, HER2-positive breast cancer

## Abstract

In current clinical practices, up to 27% of all breast cancer patients receive neoadjuvant chemotherapy. High pathological complete response rate is frequently associated with tumor-infiltrating lymphocytes. Additionally, circulating immune cells are also often linked to chemotherapy response.

We performed a retrospective analysis on a cohort of 112 breast cancer patients (79 triple-negative, 33 hormone receptor-negative/HER2-positive) treated with standard neoadjuvant chemotherapy. Eosinophil and lymphocyte counts were collected from whole blood at baseline and during follow-ups and their associations with pathological complete response, relapse, disease-free and breast cancer-specific survival were analyzed.

We observed a higher pathological complete response rate in patients who presented at baseline a relative eosinophil count ≥ 1.5% (55.6%) than in those with a relative eosinophil count < 1.5% (36.2%)(*p* = 0.04). An improvement in breast cancer-specific survival in patients with high relative eosinophil count (*p* = 0.05; HR = 0.336; 95% CI = 0.107–1.058) or with high relative lymphocyte count (threshold = 17.5%, *p* = 0.01; HR = 0.217; 95% CI = 0.060–0.783) were also observed. Upon combining the two parameters into the eosinophil x lymphocyte product with a threshold at 35.8, associations with pathological complete response (*p* = 0.002), relapse (*p* = 0.028), disease-free survival (*p* = 0.012) and breast cancer-specific survival (*p* = 0.001) were also recorded.

In conclusion, the relative eosinophil count and eosinophil x lymphocyte product could be promising, affordable and accessible new biomarkers that are predictive for neoadjuvant chemotherapy response and prognostic for longer survival in triple-negative and hormone receptors-negative/HER2-positive breast cancers. Confirmation of these results in a larger patient population is needed.

## INTRODUCTION

Breast cancer is the most common cancer and the leading cause of cancer-related death in women worldwide, with 1.67 million new cases and 522,000 deaths each year [1]. Early breast cancer is usually treated with surgery, radiotherapy and adjuvant systemic therapy. Recently, neoadjuvant treatment has become the main strategy to turn inoperable tumors into operable tumors and to allow for more frequent conservative breast surgery. Overall, approximately 7–27% of all new breast cancers in high-income countries are treated with neoadjuvant chemotherapy (NAC) treatment [2].

Pre-operative treatment allows rapid assessment of drug efficacy, and the pathological complete response (pCR) is commonly used as the endpoint for NAC trials. Evidence supporting the association between pCR and survival benefit has been previously demonstrated, especially for aggressive tumors such as triple-negative breast cancer (TNBC) and hormone receptor-negative/HER2-positive (HR−/HER2+) cancer [3–6]. Indeed, higher pCR rates were observed for TNBC and HR−/HER2+ breast cancers compared to luminal subtypes. Moreover, such a higher pCR rate was also associated with a long-term benefit [4, 7, 8].

There are currently no known factors predicting chemotherapy sensitivity in breast cancer. Recent reports have suggested a role for the immune system in chemotherapy response. Indeed, some studies demonstrated an association between tumor-infiltrating lymphocytes (TILs) and chemotherapy response [9–11]. In particular, TNBC and HR−/HER2+ breast cancers showed increased TIL scores compared to HR-positive breast cancers, and a high TIL score was associated with a higher pCR rate [8]. Most likely, the distinct TIL subpopulations contribute in a different manner in treatment response. As a matter of fact, Ladoire and colleagues described that pCR was associated with Treg depletion and an increase in the number of CD8+ T cells [12]. Furthermore, chemotherapy sensitivity and cancer outcomes both appear to be affected by circulating immune cells, including neutrophils, lymphocytes and eosinophils [13–17].

Bearing in mind the possible impact of the immune system on chemotherapy response, the aim of the current study was to analyze the potential use of pretreatment circulating eosinophil counts as a biomarker of therapy response in TNBC and HR−/HER2+ breast cancers treated with NAC.

## RESULTS

### Patient characteristics and treatment

Overall, 112 early breast cancer patients treated with NAC at the University Hospital of Liege (CHU Liege) between December 2005 and November 2017 were included in the analysis. The median follow-up was 37.5 months (range 9–156 months). The median age at diagnosis was 51.5 years (range 25–78 years). Seventy-nine patients (70.5%) had a TNBC, and 33 patients (29.5%) had an HR−/HER2+ tumor. Patients were classified according to their HER2 status, Ki67 value, tumor size, lymph node status, tumor grading, histological subtype and lymphovascular invasion, as summarized in Table 1.

**Table 1:** Baseline characteristics of the 112 patients included in the analysis

	*N* of patients (tot. 112)	% of patients
Age: median 51.5 y (25–78)		
HER2		
Positive	33	29.5
Negative	79	70.5
Ki67		
< 20%	5	4.5
≥ 20%	101	90.2
NR	6	5.4
T		
0	1	0.9
1	17	15.2
2	61	54.5
3	14	12.5
4	18	16.1
NR	1	0.9
N		
0	44	39.3
1	61	54.5
2	3	2.7
3	3	2.7
NR	1	0.9
G		
1	1	0.9
2	32	28.6
3	71	63.4
4	2	1.8
NR	6	5.4
Histological subtype		
Ductal	108	96.4
Lobular	1	0.9
Other	3	2.7
Lymphovascular invasion		
Yes	31	27.7
No	58	51.8
NR	23	20.5
pCR		
Yes	51	45.5
No	61	54.5
Chemotherapy		
EC → Ptx/Txt	71	73.4
EC → CBDCA-Ptx	8	7.1
FEC → Ptx/Txt	27	24.1
Other	6	5.4
Trastuzumab		
Yes	33	29.5
No	79	70.5
Radiotherapy		
Yes	99	88.4
No	12	10.7
NR	1	0.9
Relapse		
Yes	23	20.5
No	86	76.8
NR	3	2.7

NR: not reported; T: tumor size; N: lymph node status; G: tumor grade; pCR: pathological complete response; EC: Epirubicin-Cyclophosphamide; Ptx: Paclitaxel; CBDCA: Carboplatin; FEC: 5-fluorouracil-Epirubicin-Cyclophosphamide; Txt: Docetaxel.

Seventy-one patients received Epirubicin-Cyclophosphamide followed by Paclitaxel or Docetaxel; 8 patients received Epirubicin-Cyclophosphamide followed by Carboplatin-Paclitaxel weekly; 27 patients received 5-fluorouracil-Epirubicin-Cyclophosphamide followed by Paclitaxel or Docetaxel; 2 patients received 5-fluorouracil-Epirubicin-Cyclophosphamide alone; 2 patients received Paclitaxel in monotherapy and 2 patients received Carboplatin-Paclitaxel or Docetaxel alone because of cardiac co-morbidity. Trastuzumab was administered in all 33 HER2-positive patients. Overall, 99 patients received radiotherapy.

We observed a pCR in 51 of the 112 patients (45.5%). Relapse was observed in 23 cases (20.5%), breast cancer-related death was observed in 15 patients (13.4%), and 3 patients died from other causes.

### Relative eosinophil and lymphocyte counts association with pCR

The primary endpoint of this study is to show a statistically significant association between baseline relative eosinophil count (REC) and pCR.

**Figure 1:**
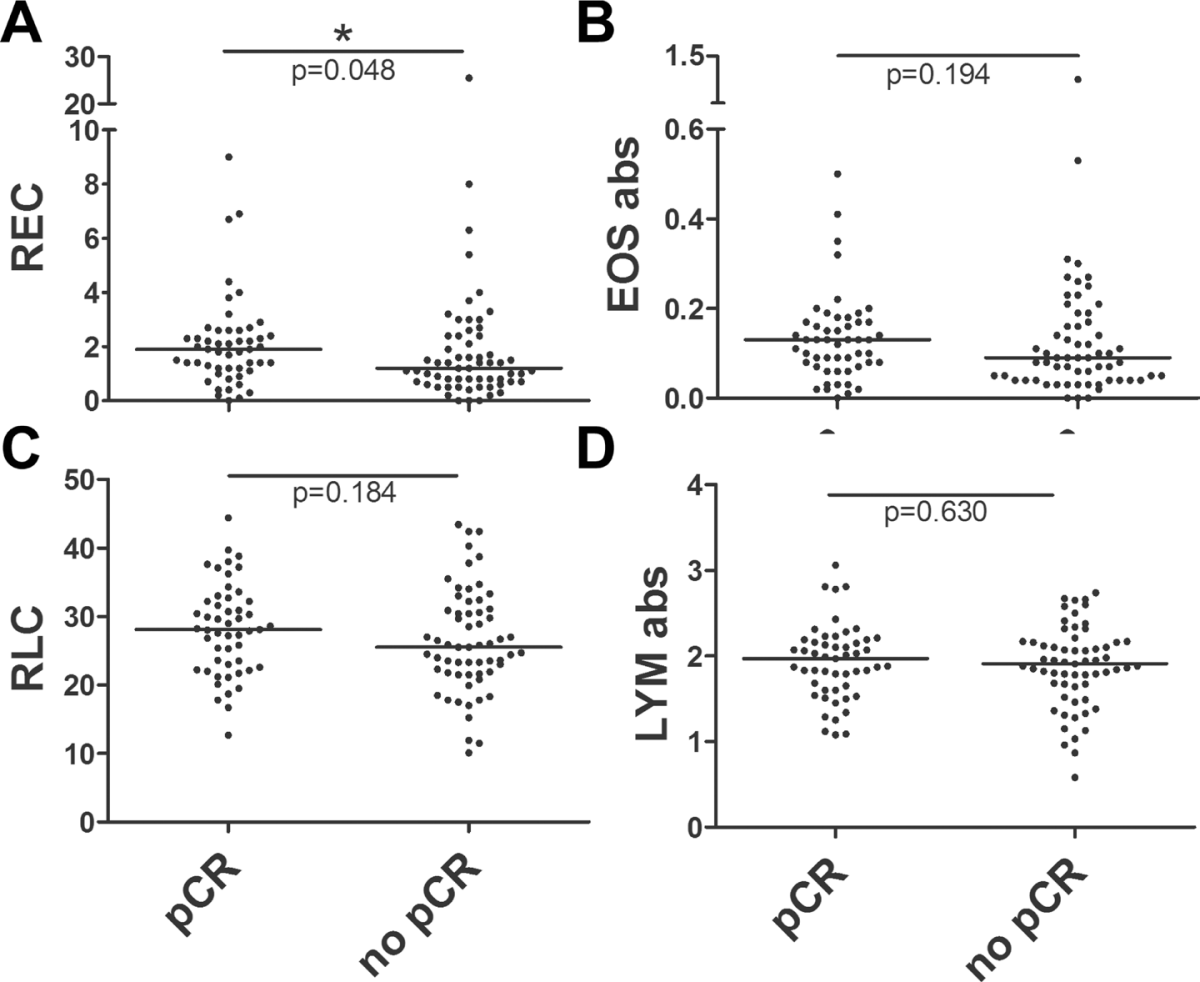
Scatter dot plots for baseline REC, RLC, absolute eosinophil and lymphocyte counts in patients with or without a pCR The comparisons between baseline distributions were calculated by the Mann-Whitney *U* test. (**A**) Scatter dot plot for baseline REC showing a median of 1.9% in patients experiencing a pCR vs 1.2% in patients without pCR (*p* = 0.048). (**B**) Scatter dot plot for baseline absolute eosinophil count showing a median of 0.13 × 10^3^/mm^3^ in patients with pCR vs 0.09 × 10^3^/mm^3^ in patients without pCR (*p* = 0.194). (**C**) Scatter dot plot for baseline RLC showing a median of 28.1% in patients experiencing a pCR vs 26.9% in patients without pCR (*p* = 0.184). (**D**) Scatter dot plot for baseline absolute lymphocyte count showing a median of 1.93 × 10^3^/mm^3^ in patients experiencing a pCR vs 1.90 × 10^3^/mm^3^ in patients without pCR (*p* = 0.630).

Patients showing a pCR after neoadjuvant treatment had a higher median REC at baseline compared to patients who did not exhibit a pCR (median REC 1.9% and 1.2% respectively; Mann-Whitney test; *p* = 0.048). No statistically significant differences were observed for baseline relative lymphocyte count (RLC) (*p* = 0.184), absolute eosinophil count (*p* = 0.194) and absolute lymphocyte count (*p* = 0.630) (Figure 1).

Patients were classified for REC and RLC, using cut-offs of 1.5% and 17.5%, respectively, as in similar studies previously published [18, 19]. Overall, 54 patients had a REC ≥ 1.5% (48.2%) and 58 had a REC < 1.5% (51.8.%), showing a significant association between high REC and pCR in univariate analysis (Chi-Square test; *p* = 0.04). To explain, 30 of the 54 patients in the high REC group (55.6%) reached a pCR whereas only 21 of the 58 patients in the group with a REC < 1.5% (36.2%) reached a pCR. Out of the 105 patients included in the high RLC group, 49 patients (46.7%) experienced a pCR, while only 2 of the 7 patients in the low RLC group (28.6%) reached a pCR. This association between pCR and RLC was not statistically significant (*p* = 0.352).

To perform multivariate analyses, univariate analyses were first calculated for all binary variables (Chi-Square test) and continuous variables (Mann-Whitney *U* test) presented in Table 2. Next, a multiple regression analysis was performed using the variables with a univariate *p* value < 0.2. The results are shown in Table 2. We observed that a greater T stage is associated with a lower rate of pCR (OR = 3.286, 95% CI = 1.673–6.453; *p* = 0.001), while a high REC is predictive of pCR (OR = 0.343, 95% CI = 0.130–0.906; *p* = 0.031).

**Table 2:** Univariate and multivariate analysis for baseline circulating eosinophils and lymphocytes in association with tumoral response to neoadjuvant chemotherapy

	Chi-Square *p*-value	Chi-Square Standard deviation	Mann-Whitney *p*-value	Multivariate analysis *p*-value	Multivariate analysis OR (95% CI)
Baseline RLC (cut-off 17.5%)	0.352	0.243	−	−	−
Baseline RLC	−	−	0.184^†^	0.643	−
Baseline absolute LYM	−	−	0.719	−	−
Baseline REC (cut-off 1.5%)	0.04^† *^	0.502	−	0.031^*^	0.343 (0.130–0.906)
Baseline REC	−	−	0.048^*^	−	−
Baseline absolute EOS	−	−	0.194^†^	0.516	−
Ki67 %	−	−	0.685	−	−
Ki67 (cut-off 20%)	0.436	0.315	−	−	−
HER2	0.216	0.458	−	−	−
Tumor size	−	−	0.009^*^	−	−
T	0.004^†*^	0.946	−	0.001^*^	3.286 (1.673–6.453)
N	0.384	0.660	−	−	−
G	0.239	0.520	−	−	−
Histology	0.198^†^	0.281	−	0.417	−
Lymphovascular invasion	0.079^†^	0.479	−	0.806	−
Type of chemotherapy	0.171^†^	1.035	−	0.724	−
Age at diagnosis	−	−	0.171^†^	0.212	−
Age (cut-off 50 y)	0.411	0.497	−	−	−

^†^variable included in multiple regression analysis; ^*^p value statistically significant

RLC: relative lymphocyte count; LYM: lymphocytes; REC: relative eosinophil count; EOS: eosinophils; T: T stage; N: lymph node status; G: grading.

Results of the univariate analysis performed by the Chi-Square test (discrete variables) or Mann-Whitney *U* test (continuous variables) for pCR using as variables the baseline relative lymphocyte and eosinophil counts, the baseline absolute lymphocyte and eosinophil counts, the Ki67, the HER2 status, the tumor size, the lymph node status, the tumor grade, the histological type, the lymphovascular invasion, the type of chemotherapy and the age at diagnosis. Multivariate analysis was performed by binary logistic regression including only the variables with a *p* value < 0.2 in the univariate test.

The primary end-point of the study was thus reached with a statistically significant positive association between REC and pCR, both in univariate or multivariate analyses.

### Relative eosinophil and lymphocyte counts association with relapse

We did not observe any statistically significant differences in the frequency of relapse between the low and high REC and RLC groups. We observed incidences of relapse of 16.7% and 24.1% in the groups with REC ≥ 1.5% and REC < 1.5%, respectively (*p* = 0.328). Considering the lymphocytes, we observed an incidence of relapse of 19.1% in the group with RLC ≥ 17.5%, whereas 42.9% of the group with RLC < 17.5% (*p* = 0.131) exhibited relapse. In a multiple regression analysis, performed with the variables showing a *p* value < 0.2 in univariate analysis, we observed a significant association with relapse for lymphovascular invasion (OR = 4.052, 95% CI = 1.255–13.083; *p* = 0.019) and for N stage (OR = 2.423, 95% CI = 1.201–4.886; p = 0.013) (Table 3).

**Table 3:** Univariate and multivariate analysis for baseline circulating eosinophils and lymphocytes in association with relapse

	Chi-Square *p*-value	Chi-Square Standard deviation	Mann-Whitney *p*-value	Multivariate analysis *p*-value	Multivariate analysis OR (95% CI)
Baseline RLC (cut-off 17,5%)	0.131	0.243	−	−	−
Baseline RLC	−	−	0.267	−	−
Baseline absolute LYM	−	−	0.048^†*^	0.303	−
Baseline REC (cut-off 1.5%)	0.328	0.502	−	−	−
Baseline REC	−	−	0.239	−	−
Baseline absolute EOS	−	−	0.267	−	−
Ki67 %	−	−	0.141^†^	0.403	−
Ki67 (cut-off 20%)	0.486	0.315	−	−	−
HER2	0.909	0.458	−	−	−
Tumor size	−	−	0.858	−	−
T	0.137^†^	0.946	−	0.320	−
N	0.031^†*^	0.660	−	0.013^*^	2.423 (1.201–4.886)
G	0.520	0.520	−	−	−
Histology	0.111^†^	0.281	−	0.303	−
Lymphovascular invasion	0.003^†*^	0.479	−	0.019^*^	4.052 (1.255–13.083)
Type of chemotherapy	0.664	1.035	−	−	−
Radiotherapy	0.714	0.312	−	−	−
Age at diagnosis	−	−	0.385	−	−−
Age (cut-off 50 y)	0.589	0.497	−	−	−

^†^variable included in multiple regression analysis; ^*^p value statistically significant

RLC: relative lymphocyte count; LYM: lymphocytes; REC: relative eosinophil count; EOS: eosinophils; T: T stage; N: lymph node status; G: grading.

Results of the univariate analysis performed by the Chi-Square test (discrete variables) or Mann-Whitney *U* test (continuous variables) for relapse using as variables the baseline relative lymphocyte and eosinophil counts, the baseline absolute lymphocyte and eosinophil counts, the Ki67, the HER2 status, the tumor size, the lymph node status, the tumor grade, the histological type, the lymphovascular invasion, the type of chemotherapy, the radiotherapy and the age at diagnosis. Multivariate analysis was performed by binary logistic regression including only the variables with a *p* value < 0.2 in the univariate test.

### Relative eosinophil and lymphocyte counts association with survival

**Figure 2:**
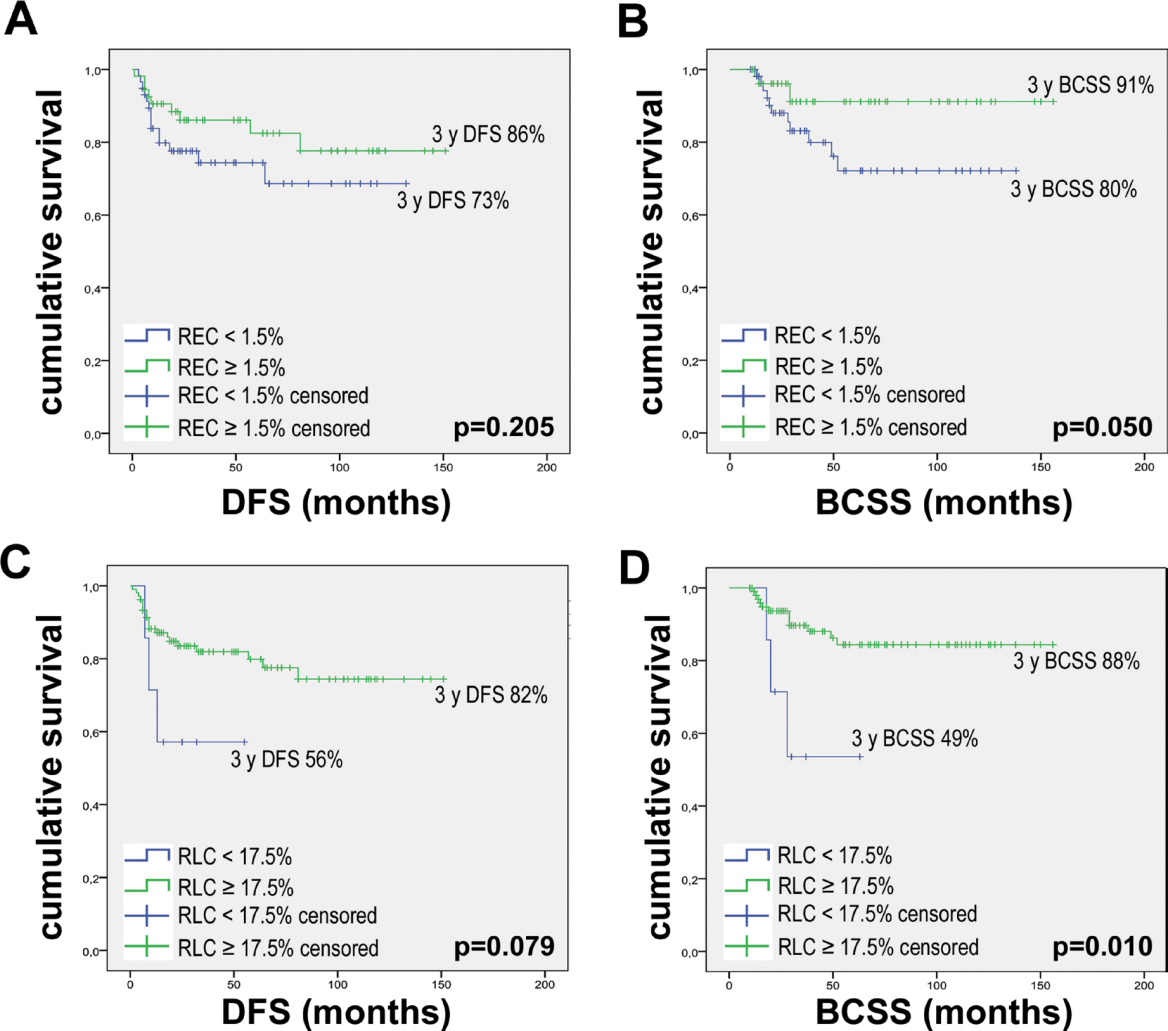
Kaplan Meier curves for DFS and BCSS according to baseline REC and RLC Kaplan Meier curves were drawn using: (**A**) REC baseline with 1.5% threshold and DFS. (**B**) REC baseline with 1.5% threshold and BCSS. (**C**) RLC baseline with 17.5% threshold and DFS. (**D**) RLC baseline with 17.5% threshold and BCSS. *P* values were calculated with the Log-Rank test.

Disease-free survival (DFS) and breast cancer-specific survival (BCSS) were analyzed with respect to REC and RLC using the same thresholds mentioned above. A 3-year DFS rate of 86% was observed for patients with REC ≥ 1.5%, whereas the DFS rate for patients with REC < 1.5% was 73% (*p* = 0.205; HR = 0.585, 95% CI = 0.252–1.358) (Figure 2A). For BCSS, we observed a statistically significant benefit for patients with higher REC, with a 3-year BCSS rate of 91% for patients with REC ≥ 1.5% compared to 80% for patients with REC < 1.5% (*p* = 0.050; HR = 0.336, 95% CI = 0.107–1.058) (Figure 2B). Likewise, we observed 3-year DFS rates of 82% and 56% for patients with RLC ≥ 17.5% and < 17.5%, respectively (*p* = 0.079; HR = 0.351, 95% CI = 0.102–1.200) and 3-year BCSS rates of 88% and 49% for high and low RLC, respectively (*p* = 0.010; HR = 0.217, 95% CI = 0.060–0.783) (Figure 2C and 2D). We chose to present the results according to cut-offs previously used in the literature for other diseases to standardize data reporting [18, 19]. However, the two thresholds used appear to be suboptimal cut-offs for separating the survival curves in breast cancer patients (Supplementary Figure 1). In fact, as shown in the Supplementary Materials, it is possible to maximize the results by calculating the cut-offs using the 3 ROC curves for pCR, relapse and breast cancer-specific death. We hypothesized that the best cut-offs for REC and RLC can be calculated as the mean values of the 3 Yunden indexes calculated on the 3 ROC curves: 1.32% for REC and 24.68% for RLC (Supplementary Figure 2).

### Eosinophil-lymphocyte product (ELP)

We combined REC with RLC by calculating the product of their respective relative values, which we defined as the eosinophil-lymphocyte product (ELP). We first calculated the Receiver Operating Characteristic (ROC) curves for pCR, relapse and death. Next, the respective Yunden indexes were calculated for each event. The mean value between the three found of 38.5 was selected as the cut-off for our analysis (Supplementary Figure 2).

Overall, 55 patients (49.1%) were classified in the group with ELP < 35.8, and 57 patients (50.9%) were classified in the group with ELP ≥ 35.8.

Among the patient groups, a pCR was observed in 59.6% of patients with ELP ≥ 35.8 and in 30.9% of patients with ELP < 35.8, with a statistically significant association between these factors (Chi Square; *p* = 0.002). In multivariate analysis, ELP appears to be predictive for pCR (OR = 0.249, 95% CI = 0.092–0.669; *p* = 0.006), while a greater T stage is associated with a lower rate of pCR occurrence (OR = 3.118, 95% CI = 1.593–6.101; *p* = 0.001) (Table 4).

**Table 4:** Univariate and multivariate analysis for ELP in association with tumoral response to neoadjuvant chemotherapy

	Chi-Square *p*-value	Chi-Square Standard deviation	Mann-Whitney *p*-value	Multivariate analysis *p*-value	Multivariate analysis OR (95% CI)
ELP (cut-off 35.8)	0.002 ^†*^	0.285	−	0.006^*^	0.249 (0.092−0.669)
ELP	−	−	0.025^*^	−	−
Ki67 %	−	−	0.685	−	−
Ki67 (cut-off 20%)	0.315	0.315	−	−	−
HER2	0.216	0.458	−	−	−
Tumor size	−	−	0.009^*^	−	−
T	0.004^† *^	0.946	−	0.001^*^	3.118 (1.593−6.101)
N	0.384	0.660	−	−	−
G	0.239	0.520	−	−	−
Histology	0.198^†^	0.281	−	0.374	−
Lymphovascular invasion	0.079^†^	0.479	−	0.822	−
Type of chemotherapy	0.171^†^	1.035	−	0.617	−
Age at diagnosis	−	−	0.171^†^	0.102	−
Age (cut-off 50 y)	0.411	0.497	−	−	−

^†^variable included in multiple regression analysis; ^*^p value statistically significant

ELP: eosinophil-lymphocyte product; T: T stage; N: lymph node status; G: grading.

Results of the univariate analysis performed by the Chi-Square test (discrete variables) or Mann–Whitney *U* test (continuous variables) for pCR using as variables the ELP, the Ki67, the HER2 status, the tumor size, the lymph node status, the tumor grade, the histological type, the lymphovascular invasion, the type of chemotherapy and the age at diagnosis. Multivariate analysis was performed by binary logistic regression including only the variables with a *p* value < 0.2 in the univariate test.

ELP is also predictive of relapse in univariate analysis, with a rate of relapse of 12.3% in patients with ELP ≥ 35.8 and 29.1% in patients with ELP < 35.8 (Chi Square; *p* = 0.028). In multivariate analysis, only nodal positivity is associated with an increased risk of relapse (OR = 2.969, 95% CI = 1.377–6.403; *p* = 0.006) (Table 5).

**Table 5:** Univariate and multivariate analysis for ELP and relapse

	Chi-Square *p*-value	Chi-Square Standard deviation	Mann-Whitney *p*-value	Multivariate analysis *p*-value	Multivariate analysis OR (95% CI)
ELP (cut-off 35.8)	0.028^†*^	0.285	−	0.054	0.274 (0.073–1.025)
ELP	−	−	0.173	−	−
Ki67 %	−	−	0.141^†^	0.341	−
Ki67 (cut-off 20%)	0.486	0.315	−	−	−
HER2	0.909	0.458	−	−	−
Tumor size	−	−	0.858	−	−
T	0.137^†^	0.946	−	0.437	−
N	0.031^†*^	0.660	−	0.006^*^	2.969 (1.377–6.403)
G	0.520	0.520	−	−	−
Histology	0.111^†^	0.281	−	0.334	−
Lymphovascular invasion	0.003^†*^	0.479	−	0.06	3.218 (0.953–10.866)
Type of chemotherapy	0.664	1.035	−	−	−
Radiotherapy	0.714	0.312	−	−	−
Age at diagnosis	−	−	0.385	−	−−
Age (cut-off 50 y)	0.589	0.497	−	−	−

^†^variable included in multiple regression analysis; ^*^p value statistically significant.

ELP: eosinophil-lymphocyte product; T: T stage; N: lymph node status; G: grading.

Results of the univariate analysis performed by the Chi-Square test (discrete variables) or Mann-Whitney *U* test (continuous variables) for relapse using as variables the baseline ELP, the Ki67, the HER2 status, the tumor size, the lymph node status, the tumor grade, the histological type, the lymphovascular invasion, the type of chemotherapy, the radiotherapy and the age at diagnosis. Multivariate analysis was performed by binary logistic regression including only the variables with a *p* value < 0.2 in the univariate test.

**Figure 3:**
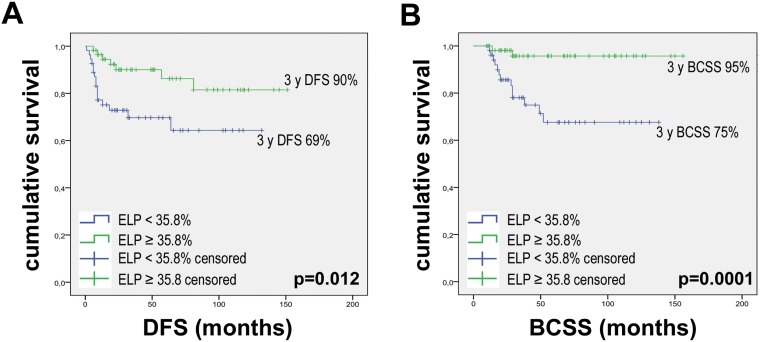
Kaplan–Meier curves for DFS and BCSS according to ELP Kaplan–Meier curves were drawn using: (**A**) ELP baseline with 35.8% threshold and DFS. (**B**) ELP baseline with 35.8% threshold and BCSS. *P* values were calculated with the Log-Rank test.

ELP is also a prognostic factor for survival, with 3-year DFS rates of 90% and 69% for patients with high and low ELP, respectively (*p* = 0.012; HR = 0.337, 95% CI = 0.138–0.823). Further, we observed 3-year BCSS rates of 95% and 75% for patients with high and low ELP, respectively (*p* = 0.001; HR = 0.129, 95% CI = 0.029–0.573) (Figure 3).

### Relative eosinophil and lymphocyte count during follow-up

**Figure 4:**
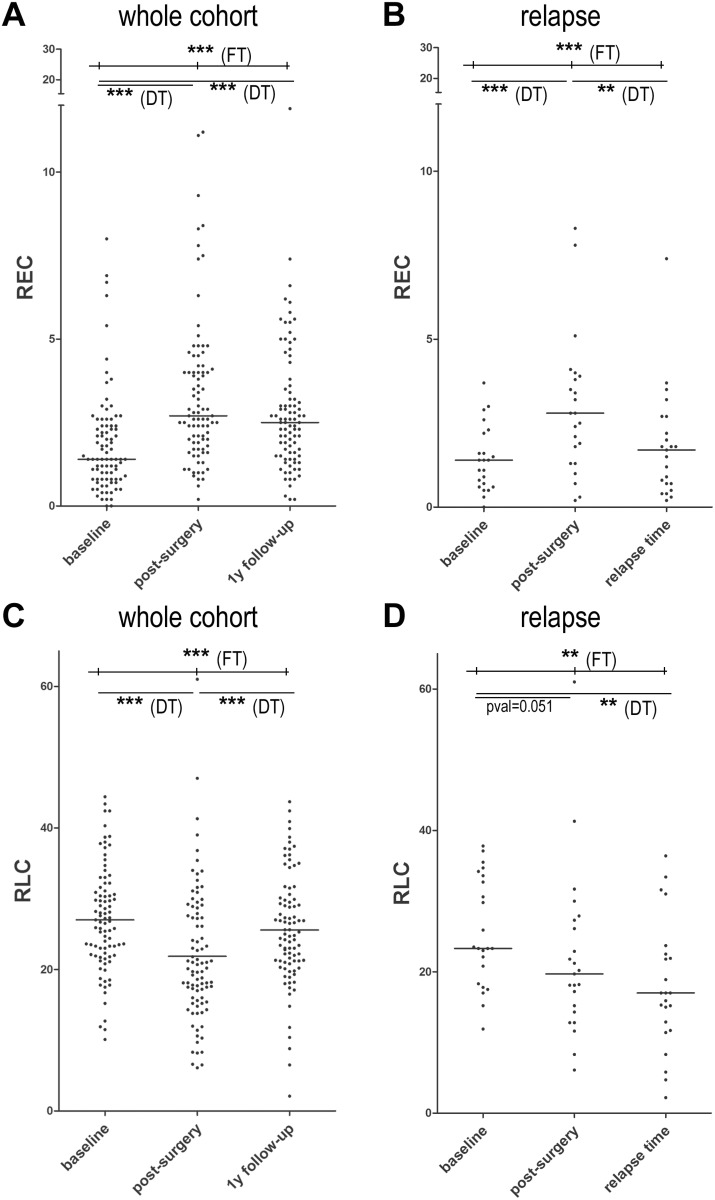
Scatter dot plots for REC and RLC after surgery, after 1 year of follow-up and at relapse (**A**) Scatter dot plot for baseline REC in the entire cohort showing a median of 1.4% at baseline vs 2.7% after surgery and 2.5% after 1-year of follow-up. (**B**) Scatter dot plot for baseline REC in 23 patients experiencing a relapse showing a median of 1.4% at baseline vs 2.8% after surgery and 1.7% at relapse. (**C**) Scatter dot plot for baseline RLC in the entire cohort showing a median of 26.75% at baseline vs 20.15% after surgery and 24.90% after 1-year of follow-up. (**D**) Scatter dot plot for baseline RLC in 23 patients experiencing a relapse showing a median of 23.3% at baseline vs 19.7% after surgery and 17% at relapse. The comparisons between baseline REC and RLC values in the 3 conditions were performed by Friedman tests (FT - upper line of *p* values) followed by Dunn post-hoc tests to compare conditions by pairs (DT - lower lines of *p* values). The corresponding significant *p* values are reported in each panel. ^***^= pval ≤ 0.0001; ^**^= pval ≤ 0.001.

At time points directly following surgery and during follow-up, we observed variations in lymphocyte and eosinophil counts. In particular, we observed an increase in REC from 1.4% at baseline to 2.7% after surgery and 2.5% after 1-year of follow-up (Figure 4A). Conversely, we observed a decrease in RLC from 26.75% to 20.15% after surgery, but no difference between baseline and 1-year follow-up (24.90%) (Figure 4C).

In the 23 patients who experienced a relapse, we observed statistically significant variations in REC from 1.4% at baseline to 2.8% after surgery, and 1.7% at relapse (Figure 4B). No statistically significant variation was detected for RLC at relapse compared to the post-surgery time point (Figure 4D). Similar results were obtained from the absolute values of circulating eosinophils and lymphocytes (data not shown).

Considering the values recorded after surgery, we observed a trend of association between REC and relapse, with a relapse rate of 18% in patients with REC ≥ 1.5% and 37.5% in patients with REC < 1.5% (Chi Square; *p* = 0.078). RLC is not associated with relapse (*p* = 0.574).

**Figure 5:**
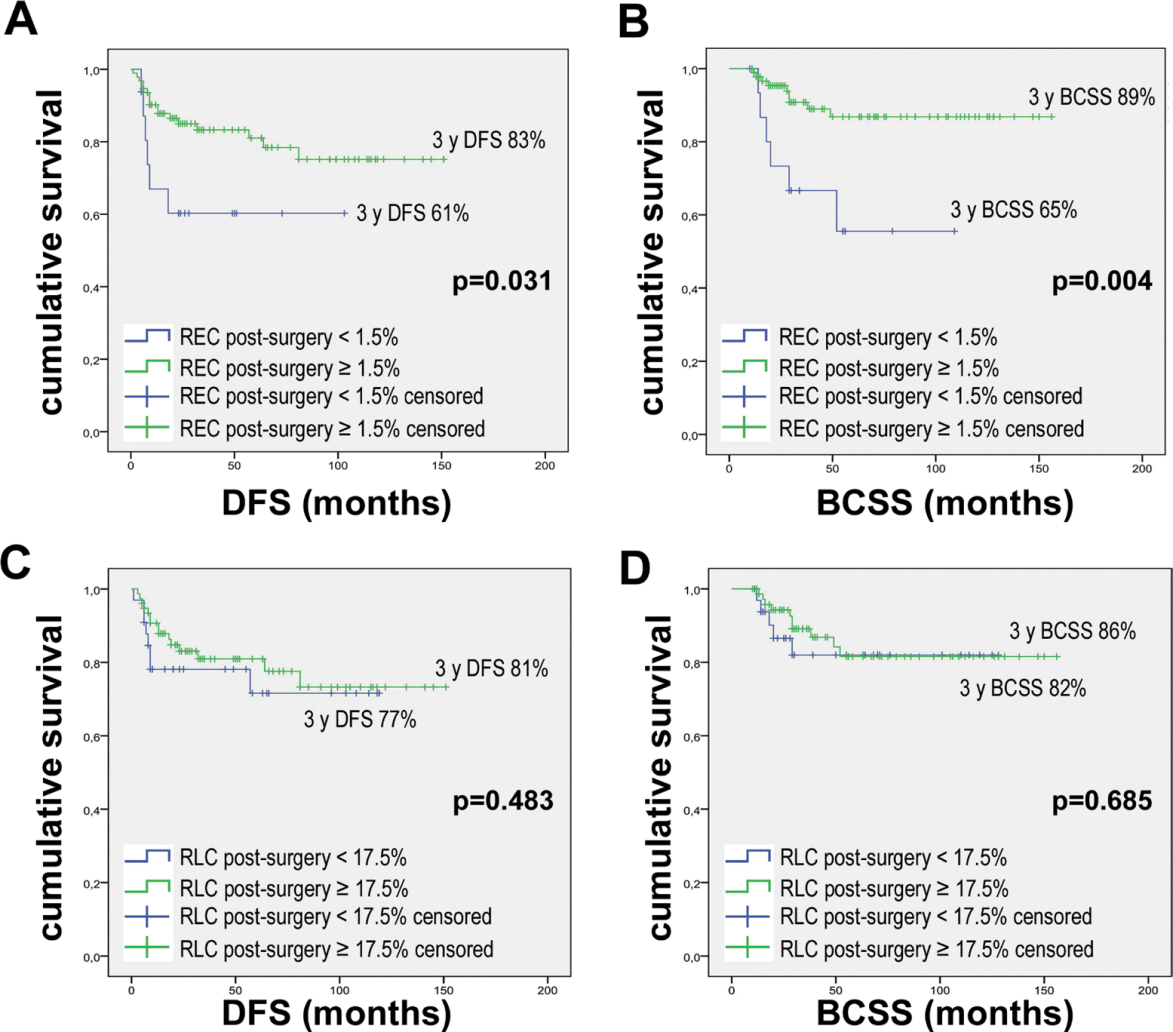
Kaplan-Meier curves for DFS and BCSS according to post-surgery REC and RLC Kaplan Meier curves were drawn using: (**A**) REC post-surgery with 1.5% threshold and DFS. (**B**) REC post-surgery with 1.5% threshold and BCSS. (**C**) RLC post-surgery with 17.5% threshold and DFS. (**D**) RLC post-surgery with 17.5% threshold and BCSS. *P* values were calculated with Log-Rank test.

Moreover, we observed a survival benefit in patients with high REC compared to those with low REC, with 3-year DFS rates of 83% and 61% for high and low REC patients, respectively (*p* = 0.031; HR = 0.371, 95% CI = 0.145–0.950). We further observed 3-year BCSS rates of 89% and 65% for high and low REC patients, respectively (*p* = 0.004; HR = 0.243, 95% CI = 0.086–0.683). Conversely, post-surgery RLC is not associated with DFS (*p* = 0.483; HR = 0.737, 95% CI = 0.312–1.710) or BCSS (*p* = 0.685; HR = 0.801, 95% CI = 0.274–2.346) (Figure 5).

## DISCUSSION

In the current study, we analyzed the effect of eosinophils and lymphocytes on response to neoadjuvant chemotherapy and survival in HR-/HER2+ breast cancer. We observed a significant association between higher REC and pCR, but not between RLC and pCR. In terms of survival, we observed a significantly longer survival period for patients with higher REC and higher RLC, with the most important differences observed using the ELP, a combined index incorporating the relative eosinophil and lymphocyte counts. Our analyses focused on HR−/HER2+ and TNBC subtypes for two reasons: first, the immune system has a key role in response to NAC; second, pCR is commonly used as a surrogate endpoint of survival for these more aggressive tumors [8, 20].

A simple blood analysis could reveal the status of the whole immune system, with circulating immune dysfunction probably linked to intra-tumoral immune inhibition or to an impaired ability of T cells to mount an anti-tumor immune response [21]. Different studies have demonstrated the association between blood cell counts and outcomes in several cancer types. In breast cancer, pretreatment lymphopenia was associated with poor survival and was predictive of tumor recurrence, and a higher absolute lymphocyte count predicted lower mortality in TNBC [13, 14, 22]. Similarly, the neutrophil/lymphocyte ratio (NLR) and the platelet/lymphocyte ratio (PLR) were described to have prognostic value in breast cancer [21–31]. To the best of our knowledge, circulating eosinophil counts have been reported by two studies in the literature of breast tumors. Gunduz and colleagues observed a survival benefit for patients with lower baseline eosinophil counts in a cohort of 62 HER2+ breast cancers treated with adjuvant trastuzumab [15]. Conversely, Ownby and colleagues described a positive association between high baseline eosinophil counts and lower recurrence rates (2-year DFS rate, 21 ± 2% vs 34 ± 8%, *p* < 0.02) in 419 patients, of all subtypes [14]. Better known is the impact of peripheral eosinophil count in melanoma patients treated with immunotherapy. Associations linking both high baseline eosinophil counts and increased counts during treatment, with both improved treatment response and increased survival rates, were observed [18, 19, 32–34]. Additionally, one report of an immunotherapy-induced increase in eosinophil count was published for lung cancer patients, but no efficacy data were presented [35].

Only one study analyzing circulating lymphocytes after neoadjuvant chemotherapy and surgery in breast cancer patients was found in the literature, while no data about eosinophils were reported. Beitsch and colleagues studied 15 early breast cancer patients, observing an impairment of natural killer (NK) cell activity with neither decreased NK cells number, nor changes in the percentage of T helper and suppressor cells [36]. In our study, we observed a decrease in circulating lymphocyte numbers after primary treatment without significant variation at relapse. No significant impact on survival for post-surgery RLC was detected. Conversely, we observed an increase in circulating eosinophil number after surgery and a significant reduction at relapse. Higher post-surgery REC was shown to be prognostic for DFS and BCSS, in accordance with baseline values. This observation suggests a role of tumor presence on peripheral eosinophil count, that could be linked to tumor infiltration by eosinophils or to a modification of tumor-induced immune cell differentiation.

Eosinophils are considered multifunctional cells that act by inducing tumor lysis or modulating immune responses [37]. Recent studies suggested that tumor-infiltrating eosinophils secrete chemoattractant cytokines that guide CD8+ T cells into cancer tissue and induce normalization of the tumor vasculature [38]. Moreover, eosinophils act as antigen-presenting cells (APCs) via surface expression of the major histocompatibility complex I and II (MHC) receptors or by directly stimulating T cells through the expression of costimulatory molecules such as CD86, CD40, CD40L and CD28 [37, 39, 40]. Although some studies have analyzed the role of tumor-associated tissue eosinophilia (TATE), less is known about the role of circulating eosinophils, and their trafficking between the circulating and tumoral compartments. However, peripheral eosinophils and a combined index between eosinophils and lymphocytes, such as the proposed ELP, could be considered biomarkers predictive of NAC response and prognostic for increased survival. In the current study, the number of patients, the short follow-up time and the subsequent number of events registered are important limitations to draw definitive conclusions. Additional studies must be performed to confirm our results and to understand the mechanism by which circulating eosinophils affect patient prognosis, with the goal of exploiting this natural anticancer mechanism to personalize patient treatment. It is worth noting that the low number of events reported did not fulfill the requirements for the parametric Cox regression. Therefore, a non-significant value was obtained while calculating the risk of death between the low and high eosinophil groups. Such results underline the importance of conducting a long-term follow-up to measure the association between eosinophil counts and BCSS. Nevertheless, these results are encouraging due to the survival of many patients with high eosinophil rates by the 3-year follow-up. The innovative aspect of our study is to propose a new, affordable and accessible biomarker predictive of treatment response and prognostic for survival for TNBC and HR−/HER2+ breast cancers.

## MATERIALS AND METHODS

### Patient selection and treatment

The current study is a retrospective analysis of a cohort of early breast cancer patients treated with neoadjuvant chemotherapy at CHU Liege between December 2005 and November 2017. The data cut-off is July 2018.

All selected patients were women, in good Performance Status (PS 0–2) and with adequate organ function prior to the beginning of treatment. Only HR−/HER2+ and TNBC patients were included in this analysis.

All enrolled patients received neoadjuvant chemotherapy with different drugs according to physician choice (including Epirubicin, Cyclophosphamide, Paclitaxel/Docetaxel, Carboplatinum, 5-Fluorouracil and/or Trastuzumab) and taking into account HER2 status. Chemotherapy was followed by surgery with or without radiotherapy.

The total number of circulating eosinophils and lymphocytes were assessed before starting treatment, after surgery, after 1-year of follow-up and at relapse. Cut-offs of 1.5% for REC and 17.5% for RLC were used, according to similar studies conducted in melanoma patients, which used an optimization algorithm to identify the thresholds required to obtain the most significant relation with OS through a Log-Rank test [18, 19, 41].

The pCR was evaluated on histological samples, after surgery (ypT0N0 following the AJCC-UICC classification). The follow-up was done according to standard clinical practices and the relapse was defined as evidence of disease recurrence using imaging and/or histological analyses. DFS is defined as the length of time between the end of the primary treatment and the evidence of cancer recurrence. BCSS is considered as the length of time between diagnosis and death from breast cancer.

The primary endpoint of this analysis was to evaluate the association between baseline REC and pCR. The secondary endpoints were to evaluate: the association between baseline RLC and pCR; the association between baseline and post-surgery REC or RLC and relapse; the median absolute values and median relative values of eosinophils and lymphocytes in patients with and without pCR; the variations in eosinophil and lymphocyte counts after surgery, at follow-up and at relapse; and the 3-year DFS and BCSS rates and respective HR according REC, RLC and ELP.

This study was conducted in accordance with the Declaration of Helsinki, and the institutional ethics committee approved the protocols.

### Statistical analysis

Statistical analyses and graphs were done using IBM SPSS Statistic v24 and GraphPad Prism 5.

Univariate analyses: Pearson Chi-Squared tests were used to calculate the associations between tumor response to neoadjuvant chemotherapy and relapse with the following discrete variables: baseline and post-surgery REC (threshold = 1.5%) and RLC (threshold = 17.5%), baseline ELP (threshold=35.8%), HER2 status, Ki67 (threshold = 20%), tumor stage, lymph node status, tumor grade, histological type, lymphovascular invasion, age at diagnosis (threshold = 50 years), types of chemotherapy and radiotherapy (only for relapse analysis). Mann–Whitney *U* tests were used to calculate the associations between tumor response to neoadjuvant chemotherapy and relapse with the following continuous variables: baseline relative lymphocyte and eosinophil counts, baseline absolute lymphocyte and eosinophil counts, ELP, tumor size, Ki67 and age at diagnosis.

Multivariate analyses: sequential binary logistic regression with forward stepwise selection of variables based on likelihood ratio were performed for both response and relapse, considering as dependent variables only the factors showing a *p* value < 0.2 in the Chi Square test or Mann-Whitney test. Some variables were tested as continuous and discrete variables (i.e. : Ki67), and when the *p* value was < 0.2 in both cases, the variable with the best *p* value was selected for multivariate analysis.

Kaplan Meier and Log-Rank tests were used to analyze survival. The Cox Regression Hazard model was used to calculate the HR for both DFS and BCSS. The 3-year DFS and BCSS were calculated from the survival tables.

To evaluate the differences in the REC and RLC distributions during follow-up, we used a Friedman test comparing the three following conditions: baseline vs post-surgery vs 1-year follow-up. For the 23 patients showing a recurrence, the same test was used to compare the REC and RLC values at baseline vs post-surgery vs relapse time. The Friedman tests were followed by Dunn multiple comparison post-hoc tests to compare all conditions by pairs.

To select the best cut-off for ELP, the ROC curves for pCR, relapse and death were drawn. The best cut-off for each variable was calculated using the Yunden index, and the threshold selected for our analysis was the mean value between the three cut-offs calculated.

## SUPPLEMENTARY MATERIALS FIGURES



## Abbreviations

APC: antigen-presenting cell
BCSS: breast cancer-specific survival
CBC: complete blood count
DFS: disease-free survival
ELP: eosinophil/lymphocyte product
G: tumor grade
HR: hormone receptor
MHC: Major Histocompatibility Complex
N: lymph nodes status
NK: natural killer
NLR: neutrophil/lymphocyte ratio
OS: overall survival
pCR: pathological complete response
PLR: platelet/lymphocyte ratio
REC: relative eosinophil count
RLC: relative lymphocyte count
ROC: receiver operating characteristic
T: tumor size
TATE: tumor-associated tissue eosinophilia
TIL: tumor-infiltrating lymphocyte
TNBC: triple-negative breast cancer.
