# Supplementary material for: Predictive and prognostic role of peripheral blood eosinophil count in triple-negative and hormone receptor-negative/HER2-positive breast cancer patients undergoing neoadjuvant treatment
Source: Oncotarget. 2018 Sep 14;9(72):33719–33. doi: 10.18632/oncotarget.26120 (PMC6154746; doi:10.18632/oncotarget.26120)
Supplement: Supplementary file 1 [file oncotarget-09-33719-s001.pdf]

Predictive and prognostic role of peripheral blood eosinophil count in triple-negative and hormone receptor-negative/HER2-positive breast cancer patients undergoing neoadjuvant treatment

SUPPLEMENTARY MATERIALS

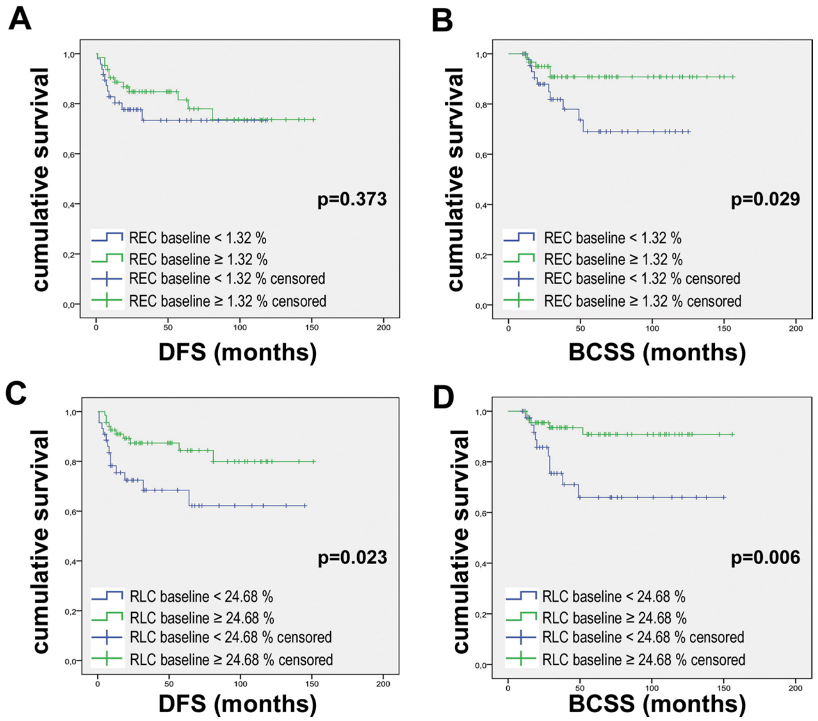

Supplementary Figure 1: Kaplan Meier curves for DFS and BCSS according to baseline REC and RLC. Kaplan Meier curves were drawn using: (**A**) REC baseline with 1,32 % as threshold and DFS; (**B**) REC baseline with 1,32 % as threshold and BCSS; (**C**) RLC baseline with 24.7 % as threshold and DFS; (**D**) RLC baseline with 24.7 % as threshold and BCSS. The thresholds were based on the mean value of the 3 Yunden indexes calculated in Supplementary Figure 2D.

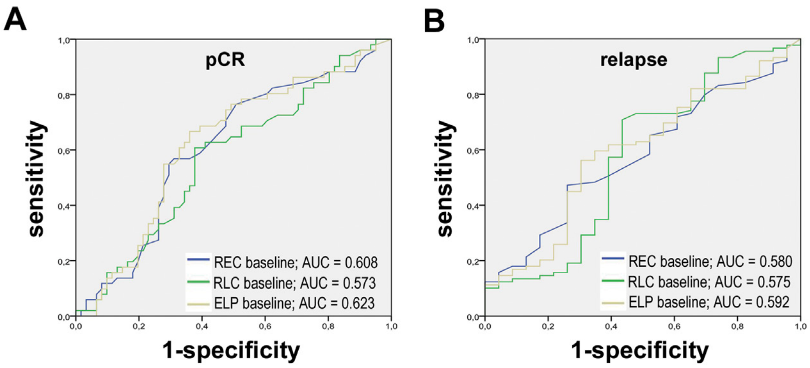

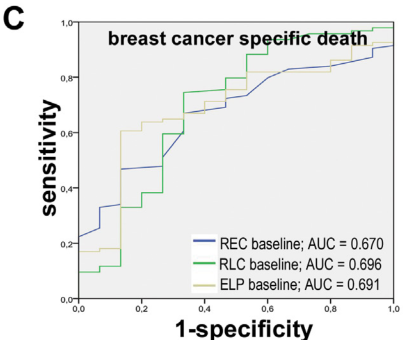

D

Yunden indexes			
	REC	RLC	ELP
pCR	1.15%	27.10%	37.24%
relapse	1.65%	23.55%	36.02%
breast cancer specific death	1.15%	23.40%	34.16%
**mean**	**1.32%**	**24.68%**	**35.81%**

Supplementary Figure 2: ROC curves and Yunden indexes for pCR, relapse and breast cancer specific death. Yunden indexes (**D**), that are maximazing the sensitivity and specificity, were calculated from the coordinates of the 3 following ROC curves: (**A**) prediction of pCR by REC baseline (blue) or by RLC baseline (green) or by ELP baseline (yellow); (**B**) prediction of relapse by REC baseline (blue) or by RLC baseline (green) or by ELP baseline (yellow); (**C**) prediction of beast cancer specific death by REC baseline (blue) or by RLC baseline (green) or by ELP baseline (yellow).
